# Decreased wrist rotation imitation abilities in children with autism spectrum disorder

**DOI:** 10.3389/fpsyt.2024.1349879

**Published:** 2024-04-18

**Authors:** Fulin Liu, Kai Qiu, Hongan Wang, Yuhong Dong, Dongchuan Yu

**Affiliations:** ^1^ Key Laboratory of Child Development and Learning Science of Ministry of Education, School of Biological Science and Medical Engineering, Southeast University, Nanjing, China; ^2^ Henan Provincial Medical Key Lab of Language Rehabilitation for Children, Sanmenxia Center Hospital, Sanmenxia, Henan, China; ^3^ Henan Provincial Medical Key Lab of Child Developmental Behavior and Learning, The Third Affiliated Hospital of Zhengzhou University, Zhengzhou, Henan, China; ^4^ Henan Provincial Engineering Research Center of Children’s Digital Rehabilitation, The Third Affiliated Hospital of Zhengzhou University, Zhengzhou, Henan, China

**Keywords:** autism spectrum disorder, meaningless gross motor imitation, wrist rotation imitation, inertial sensor, machine learning, classifier

## Abstract

**Introduction:**

While meaningless gross motor imitation (GMI) is a common challenge for children diagnosed with autism spectrum disorder (ASD), this topic has not attracted much attention and few appropriate test paradigms have been developed.

**Methods:**

The current study proposed a wrist rotation imitation (WRI) task (a meaningless GMI assignment), and established a WRI ability evaluation system using low-cost wearable inertial sensors, which acquired the simultaneous data of acceleration and angular acceleration during the WRI task. Three metrics (i.e., total rotation time, rotation amplitude, and symmetry) were extracted from those data of acceleration and angular acceleration, and then were adopted to construct classifiers based on five machine learning (ML) algorithms, including k-nearest neighbors, linear discriminant analysis, naive Bayes, support vector machines, and random forests. To illustrate our technique, this study recruited 49 ASD children (aged 3.5-6.5 years) and 59 age-matched typically developing (TD) children.

**Results:**

Findings showed that compared with TD children, those with ASD may exhibit shorter total rotation time, lower rotation amplitude, and weaker symmetry. This implies that children with ASD might exhibit decreased WRI abilities. The classifier with the naive Bayes algorithm outperformed than other four algorithms, and achieved a maximal classification accuracy of 88% and a maximal AUC value of 0.91. Two metrics (i.e., rotation amplitude and symmetry) had high correlations with the gross and fine motor skills [evaluated by Gesell Developmental Schedules-Third Edition and Psychoeducational Profile-3 (PEP-3)]. While, the three metrics had no significant correlation with the visual-motor imitation abilities (evaluated by the subdomain of PEP-3) and the ASD symptom severity [evaluated by the Childhood Autism Rating Scale (CARS)] .

**Discussion:**

The strengths of this study are associated with the low-cost measurement system, correlation between the WRI metrics and clinical measures, decreased WRI abilities in ASD, and high classification accuracy.

## 1 Introduction

Autism spectrum disorder (ASD) is a life-long neurodevelopmental disorder characterized by deficits in social-communicative functioning and the presence of repetitive and restricted behaviors, interests, and activities (1), affecting approximately 1 in 36 children in the United States (2). Although motor impairment is not currently included in the diagnostic criteria for ASD, an increasing amount of research supports that there are pervasive gross motor impairments in individuals with ASD (3–5). For instance, recent prevalence studies based on the U.S. Simons Powering Autism Research (SPARK) database (total *n*=10,234-11,814; ages 5-15 years) have verified that a majority of children (86.9%) with ASD exhibit clinically significant gross motor impairments (6).

Systematic reviews and meta-analyses (3, 7, 8) have demonstrated that the association between gross motor abilities and social deficits in ASD is robust across social skills, study methods, and participant features, though the extent to which this association arises from direct causal influences between both domains or shared underlying genetic or neurological causes still remains unknown. Additionally, gross motor deficits in ASD could contribute to the development of social impairments over time by altering the ways in which individuals with ASD perceive and interact with others (3). This implies that gross motor impairments in children with ASD may compound existing vulnerabilities in the social domain (3).

Gross motor abilities involve several domains, including locomotion, balance and posture, object control, reaching, motor control and coordination, strength and agility, imitation, and broad composite (3, 9). Gross motor imitation (GMI) represents the capacity of an individual to replicate an observed motor. It involves the ability to transform perceptual information into a gross motor copy of it, and thus is regarded as one of the most important domains of gross motor abilities (9, 10). In particular, recent studies have shown that GMI is an extraordinary ability that is fundamentally linked to the development of language, social skills, and intelligence (11, 12). It should be noted that GMI can be subdivided into meaningful and meaningless GMI, which might exhibit two different neural mechanisms (13). Unfortunately, children with ASD may experience difficulties in GMI from a very early age (14, 15). Additionally, meaningless GMI cannot rely on prior knowledge of the motors themselves and might be correlated with visual attention to movements, and thereby appears to be appropriate for use in identifying ASD (3, 13, 16). For instance, studies have shown that individuals with ASD and healthy controls differ in meaningless GMI abilities (3, 13, 16).

While meaningless GMI is a common challenge for children with ASD, this topic has not attracted much attention (3). Thus far, only a few tasks have been proposed, such as imitation of full-body postures and imitation of sinusoidal arm movements (see 3 for a review). It is important to develop some new test paradigms of meaningless GMI to reveal the imitation impairments in individuals with ASD. Remarkably, the wrist rotation imitation (WRI) has been extensively used to evaluate the functional deficits and efficacy of rehabilitation in individuals with brain injuries (17–19). Additionally, due to its ease of implementation, the WRI has also been widely used in clinical neurological functioning testing for children. The current study aimed to test whether the WRI task can be adopted as a potential tool for the screening and diagnosis of ASD. Furthermore, it attempted to explore the factors to impact the imitation performances in the WRI task.

As a recent advancement in GMI assessment technology, research has attempted to use instruments to achieve objective evaluation (20). For instance, an infrared-based motion tracking system was adopted to record and analyze the kinematics during the imitation of sinusoidal arm movements (20). Results (20) demonstrated that: (i) individuals with ASD exhibited atypical kinematics; and (ii) they did not minimize jerk to the same extent as the healthy controls did, and instead moved with greater acceleration and velocity. However, due to the complexity of human motion, this infrared-based motion tracking system often results in low accuracy of (joint) position estimation. Furthermore, this motion tracking system is relatively expensive and needs a relatively large room to operate. Hence, it is necessary to consider less expensive measurement technologies, such as the use of low-cost inertial measurement units (21).

Taken together, this study adopted a WRI task and suggested the use of low-cost wearable inertial sensors, which integrated a three-axis accelerometer and a three-axis gyroscope together, to acquire the simultaneous data of acceleration and angular acceleration during the WRI task through the Internet of Thing technique (21). The main targets of this study included that: (i) deriving a few metrics to evaluate the WRI abilities from these acceleration and angular acceleration signals; (ii) testing whether there is a significant difference between both groups (i.e., children with ASD and healthy controls) in these metrics; (iii) examining whether these metrics could predict the gross motor abilities and the symptom severity of ASD; and (iv) exploring whether these metrics (taken as features) could be adopted to construct classifiers for the ASD identification using machine learning (ML) algorithms. To illustrate our technique, we recruited 49 children with ASD and 59 age-matched typically developing (TD) children, and extracted three metrics (i.e., total rotation time, rotation amplitude, symmetry) to evaluate the WRI abilities. This study aimed to test whether the suggested three metrics may be applied to distinguish the difference between both groups (i.e., ASD and TD groups) at the group difference level, as well as the individual difference level. This study also discussed the correlation between the three metrics and gross and fine motor abilities [evaluated by Gesell Developmental Schedules-Third Edition (22–25) and Psychoeducational Profile-3 (26–28)], as well as the correlation between the three metrics and the ASD symptom severity [evaluated by the Childhood Autism Rating Scale (CARS) (29–31)].

## 2 Method

The Ethics Committee of the Sanmenxia Center Hospital gave its approval (No. 2022066) to all study protocols and research techniques, ensuring that they adhered to the World Medical Association’s Declaration of Helsinki regarding the use of humans in testing. All participating children’s parents gave their informed consent, and each participant gave their oral consent before the experiment began.

### 2.1 Participants

This study was conducted in the Sanmenxia Center Hospital between March to August in 2023. We initially recruited 58 children (aged 3.5-6.5 years) from clinical cases, who were first diagnosed with ASD using the Childhood Autism Rating Scale (CARS) (29–31) and DSM-5 (1). We also recruited 111 TD children (aged 3.5-6.5 years) from a local kindergarten, and asked their teachers to classify their intelligence levels into five levels: “excellent”, “above average”, “average”, “below average”, and “very poor”. The teachers reported that all participating children had “average” intelligence level. They further reported that all participating children had no physical or mental disorders. It should be noted that all participating children with and without ASD were native Chinese speakers and right-handed.

Exclusion criteria were as follows: (a) abnormal hearing and vision functioning; (b) children with significant wrist motor impairment (checked by neurosurgery experts); (c) preterm birth; (d) girls; (d) IQ<70, measured by the Third Editon of Wechsler Intelligence Scale for Children (WISC-III); (e) the presence of pathological conditions including ADHD, epilepsy, and Tourette syndrome; and (f) incomplete clinical data associated with evaluation processing. Based on inclusion and exclusion criteria, a total of 49 ASD children (aged 4.97 ± 0.81 years) and 59 age-matched TD children (aged 5.11 ± 0.85 years) were invited to participate the current study. Each participating child received an age-appropriate toy after completing the study.

### 2.2 Experimental procedure

**Figure 1:**
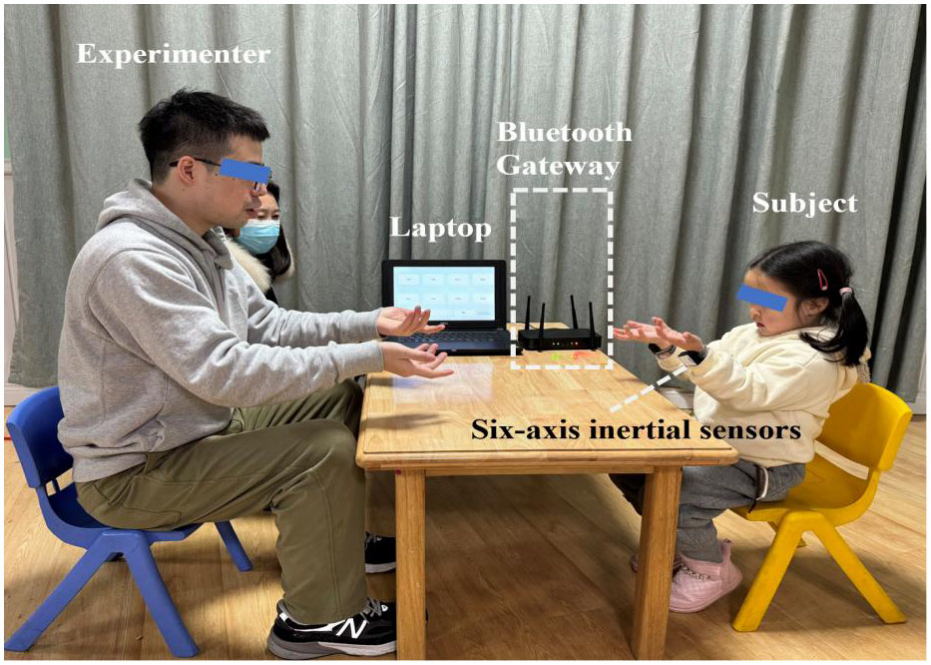
A scene showing that a subject was imitating the wrist rotation of an experimenter, where the subject wore two wearable six-axis inertial sensors on both writs to capture the three-axis accelerometer and three-axis gyroscope signals which were simultaneously sent to the laptop through the Bluetooth gateway.

This study adopted a WRI task and suggested the use of a motor evaluation system, which acquired the simultaneous data of acceleration and angular acceleration during the WRI task through the Internet of Thing technique (21). Specially, as shown in **Figure 1**, the motor evaluation system consists of two low-cost wearable inertial sensors (with embedded Bluetooth wireless communication units), a Bluetooth gateway, and a laptop, and can be adopted to capture the simultaneous data of acceleration and angular acceleration during the WRI task. Before the experiment, the experimenter helped the participants wear the two inertial sensors on both wrists and ensured that their hand movements were not restricted by the presence of the watch strap. The WRI task was completed in a quiet room, and participants were asked to sit down facing the experimenter. **Figure 1** also showed a scene that a subject was successfully imitating the wrist rotation of the experimenter.

**Figure 2:**
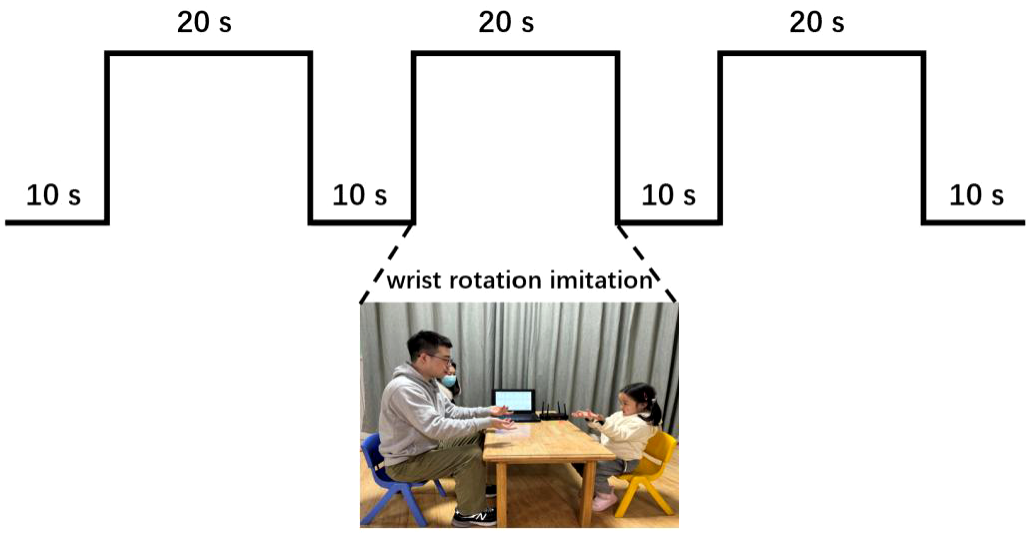
Experimental flow chart, including three blocks. Each block included a 20-second imitation duration with an interval of 10 seconds between two blocks for resting.

**Figure 3:**
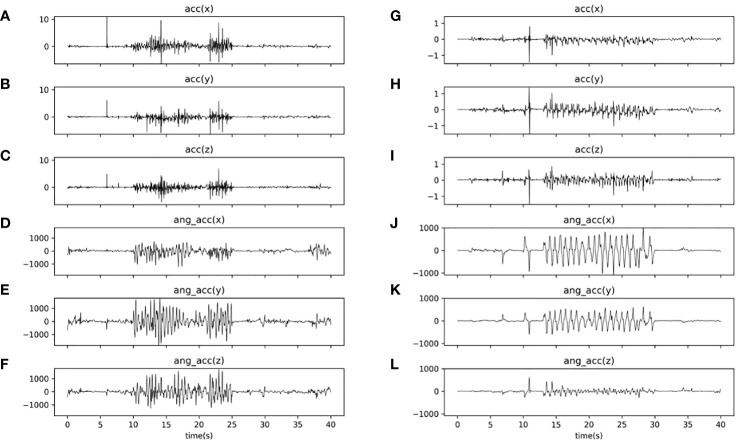
Curves plotting acceleration and angular acceleration signals of two samples (i.e., a TD subject and an ASD subject) during the WRI task. **(A–C)** three-axis (i.e., x, y, and z axis) acceleration signals of the TD sample; **(D–F)** three-axis (i.e., x, y, and z axis) angular acceleration signals of the TD sample; **(G–I)** three-axis (i.e., x, y, and z axis) acceleration signals of the ASD sample; and **(J–L)** three-axis (i.e., x, y, and z axis) acceleration signals of the TD sample.

**Figure 2** summarized the experimental flow chart. As shown in **Figure 2**, the task consisted of a total of three blocks. Each block had a 20-second imitation duration, and there was a 10-second resting period between two blocks. During each block, the participants were instructed to replicate the experimenter’s hand movement, which involved rotating his hands back and forth about twice a second. Meanwhile, six channels of real-time data, including three-axis acceleration and three-axis angular acceleration signals, were captured at a sampling rate of 50 Hz by each inertial sensor during the WRI task. Through the Bluetooth gateway, six channels of data were simultaneously delivered to the laptop (as illustrated in **Figure 1**). The entire experiment lasted approximately 100 seconds. **Figure 3** illustrated the three-axis acceleration and three-axis angular acceleration signals of two samples, who were randomly chosen from both groups (i.e., ASD and TD groups), respectively. There are visible differences in the acceleration and angular acceleration signals between both samples.

Before a formal experiment, participants were instructed to do some practice for the understanding of the whole experimental procedure. A calibration procedure would generally be conducted for each inertial sensor, which would be statistically placed on a horizontal table for 1 minute. In particular, the three-axis accelerometers and three-axis gyroscopes would be calibrated using the least squares algorithms (32) and Allan variance (33), respectively.

ASD participants were further required to attend three clinical (behavioral) evaluations utilizing the Gesell Developmental Schedules (Third Edition) (22–25), Childhood Autism Rating Scale (29–31), and Psychoeducational Profile (Third Edition) (26–28), respectively. A senior expert (with professional experience more than 10 years) carried out the clinical (behavioral) measures for all participants with ASD. The senior expert had training in administration of all tools used in this study.

### 2.3 Metrics extracted to evaluate WRI abilities

The raw six-channel data (including three-axis acceleration and three-axis angular acceleration signals) were interpolated with the piecewise cubic Hermite interpolating polynomial method and filtered with a second-order low-pass Butterworth filter (with 10 Hz cut-off), respectively. These data were further filtered using the Kalman filtering. For the data (time-series) after the aforementioned preprocessing, three metrics (i.e., total rotation time, rotation amplitude, and symmetry) were extracted to evaluate WRI abilities.

(1) Total rotation time: Total rotation time (TRT) refers to the duration time that a participant sustains to rotate his/her wrist during each block. The maximum value of TRT in the three blocks would be chosen as the TRT of an individual. The longer the total rotation time, the stronger the ability to sustain the imitation of wrist rotation.
(2) Rotation amplitude: Rotation amplitude (RoA) refers to averaged rotation angle over two adjacent peaks during each block, and can be calculated as follows:

$$RoA=\frac{1}{N-1}\sum_{i=0}^{N-1}\left|\int_{P_{i}}^{P_{i+1}}\omega (t)dt\right|$$

where *N* is the total number of sampling points; Pi is the value of the i-th peak point; and ω(t) is the angular velocity of the y-axis. The maximum value of RoA in the three blocks would be chosen as the RoA of an individual. The greater the value of RoA, the stronger the ability to track the wrist rotation of the experimenter.

(3) Symmetry: Symmetry refers to the difference of rotation motion between the right and left wrists during each block, and can be calculated as follows:

$$Symmetry={\left(1-0.7\times \frac{\left|RoA_{l}-RoA_{r}\right|}{\max(RoA_{l},RoA_{r})}-0.3\times \frac{\left|RoF_{l}-RoF_{r}\right|}{\max(RoF_{l},RoF_{r})}\right)}^{2}$$

where *RoA_l_* and *RoA_r_* are the rotation amplitude of the left and right wrists, respectively; $RoF_{l}$ and $RoF_{r}$ are the rotation frequency of the left and right wrists, respectively; and max(*k*, *h*) refers to the maximum value between the values *k* and *h*. The maximum value of symmetry in the three blocks would be chosen as the symmetry of an individual. The greater the value of symmetry, the better the consistency of rotation motion between the right and left wrists.

### 2.4 Clinical measures

All ASD participants were required to attend the following clinical evaluations, which were associated with developmental level and symptom severity of ASD.

#### 2.4.1 Gesell developmental schedules-third edition

The Chinese version of GDS-3 (22–25) is a popular and psychometrically valid scale in China, which provides a developmental profile for children aged from 16 days to 6 years old in five domains (i.e., gross motor, fine motor, adaptability, language, and personal social behavior). Gross motor (GM) domain involves an individual’s postural reaction, e.g., head stability, sitting, standing, crawling, and walking; while fine motor (FM) domain reflects an individual’s ability to grasp, manipulate objects, and coordinate hands and eyes. Adaptability (AD) domain reflects an individual’s ability to: (i) percept the organization and relationship of objects (e.g., toys); and (ii) decompose the whole of an object into its components, and recompose these components into a whole in a meaningful way. Language (LA) domain involves receptive and expressive language skills; while personal-social behavior (PSB) domain reflects an individual’s abilities in interpersonal communication and self-care.

GDS-3 offers a number of Gesell developmental standards for each domain, which involve 24 developmental milestones in 4 weeks, 8 weeks, 12 weeks, 16 weeks, 20 weeks, 24 weeks, 28 weeks, 32 weeks, 40 weeks, 44 weeks, 48 weeks, 52 weeks, 56 weeks, 15 months, 18 months, 21 months, 24 months, 30 months, 36 months, 42 months, 48 months, 54 months, 60 months, and 72 months, respectively. Furthermore, each developmental milestone contains a few items. The target of the evaluator is to check the items in each milestone one by one to see if the subjects can pass or fail. After that, the developmental age (DA) can be computed by:


$$DA=\frac{\sum_{i}W_{i}N_{i}}{\sum_{i}N_{i}}$$

where $N_{i}$ is the number of items in the milestone $W_{i}$ that the subjects can pass.

Finally, developmental quotient (DQ) score can be determined by:


DQ=100∗DA/CA

where CA is the chronological age.

In this way, GDS-3 offers a DQ score to evaluate the child’s development level for each domain, and thus retrieves five DQs (i.e., Gesell-GM-DQ, Gesell-FM-DQ, Gesell-Ad-DQ, Gesell-La-DQ, Gesell-PSB-DQ) for each individual. An individual can be classified according the following rule: a subscale DQ less than 76 points indicates a developmental delay; a quotient between 76 and 85 points is slightly below the threshold for developmental delay; and a quotient greater than or equal to 86 points indicates normal development. The reliability of GDS-3 in the Chinese population ranged from 0.90 to 0.97 for all domains (34).

#### 2.4.2 Childhood autism rating scale

This study adopted the original version (i.e., the first edition) of the CARS scale, which was a 15-iem scale and conducted by highly trained raters (29, 31). It evaluates behavior in 14 domains that are typically affected by severe autism-related issues as well as one general category of impressions of autism, with the goal of differentiating autistic children from those with other developmental disorders. The assessment was based on data gathered through a variety of techniques, including direct observation, parent interviews, and analysis of previously collected clinical data. Scores for each item range from 1 to 4, with 1 denoting behavior appropriate for the age level and 4 denoting extreme deviation from the expected behavior for the age level. According to the total CARS score (i.e., the sum of the scores for all items), each child may be labeled as “not autistic” (score below 30), “mild or moderately autistic” (scoring between 30 and 36), or “severely autistic” (score above 36). The reliability and validity of CARS scale in the Chinese population were 0.73 and 0.97, respectively (30).

#### 2.4.3 Psychoeducational profile-3

The PEP-3 (26) is a norm-referenced scale measuring development and maladaptive behavior in children with ASD between the developmental ages of 6 months to 7 years (26). The PEP-3 includes two major components: the Performance Test and the Caregiver Report. This study only considered six subsets in the Performance Test, including Cognitive Verbal/Preverbal (PEP3-CVP), Expressive Language (PEP3-EL), Receptive Language (PEP3-RL), Fine Motor (PEP3-FM), Gross Motor (PEP3-GM), and Visual-Motor Imitation (PEP3-VMI) skills. It should be noted that these six subsets are adopted to measure the developmental level of children. Previous studies have shown that the Chinese version of the PEP-3 has good psychometric properties and Cronbach’s alpha coefficients for all subscales were above 0.80 (27, 28).

### 2.5 Statistical analysis

Statistical analyses in this study mainly involved three aspects. Firstly, after confirming that our data failed to pass the normality test and variance homogeneity test, we performed a series of non-parametric two-way analysis of variance (ANOVA) for the suggested three metrics, where two factors were age and population-type. In addition, for post-hoc pairwise comparisons, we utilized the ART-C algorithm (35) with the Bonferroni correction applied to *p*-values to control the false discovery rate.

Secondly, we adopted the software toolbox, Weka 3.8.6 (36), to build ASD classifiers by five wildly-used ML algorithms, including k-nearest neighbors (KNN), linear discriminant analysis (LDA), naive Bayes (NB), support vector machines (SVM), and random forests (RF). The input variables included in the classifiers were the three rotation metrics (i.e., total rotation time, rotation amplitude, and symmetry), and the output variable was the group coding (0 and 1 were corresponding to TD and ASD children, respectively). To construct ML-based classifiers, we randomly divided the collecting data into training and testing datasets in a ratio of 90% to 10%, and determined the optimal parameters of each learning model by repeating 10 cross validation and grid search methods. The predictive performances of each classifier were evaluated using the receiver operating characteristic curve (ROC) analysis, which used three important indices, i.e., sensitivity, specificity and accuracy (37). Sensitivity and specificity further formed an integral index, i.e., area under the curve (AUC), which can be applied to evaluate model prediction performance (38, 39).

Thirdly and finally, for ASD participants, this study sought to explore the correlation between the suggested three metrics and clinical (behavioral) measures (including GDS-3, PEP-3, and the CARS scores) by Spearman’s correlation analysis without the control of false discovery rate.

All statistical analysis above was conducted with R language (version 4.3.1) (35, 40–42) and the significance level α was set at 0.05.

## 3 Results

### 3.1 Demographic information

This study involved 108 children, including 34 children (with 15 ASD children) aged 3.5-4.5 years, and 35 children (with 15 ASD children) aged 4.5-5.5 years, and 39 children (with 19 ASD children) aged 5.5-6.5 years old. **Table 1** summarizes detailed demographic features. The chi-square test results showed that the both groups (i.e., ASD and TD groups) were numerically matched in the three age groups (*χ^2^ = *0.29, *p*=0.87).

**Table 1:** Demographic information.

Age Groups	Population Groups		
	ASD(*n*)	TD(*n*)	Total(*n*)
3.5-4.5 yr. (48.9 ± 3.82 month)	15	19	34
4.5-5.5 yr. (59.3 ± 3.21 month)	15	20	35
5.5-6.5 yr. (71.5 ± 3.82 month)	19	20	39
Total	49	59	108
*χ^2^, p*	0.29, 0.87		–

### 3.2 WRI features

**Figure 4:**
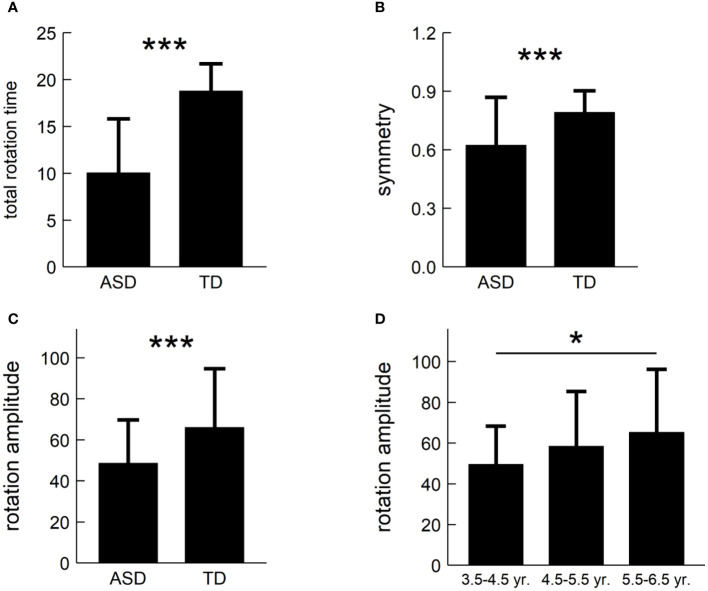
Post-hoc pairwise comparisons of the three metrics: **(A)** total rotation time; **(B)** symmetry; and **(C)** rotation amplitude. **(D)** illustrating age difference in rotation amplitude. *: *p*<0.05, ***: *p*<1×10^-3^.

For each of the suggested three metrics, we conducted a two-factor non-parametric ANOVA to test the main effects and their interaction. **Figure 4** summarized our results and showed that: (i) the main effect of population-type was significant for total rotation time, rotation amplitude, and symmetry (*F*: 70.87, 14.72, 15.49; *p*’*s*<1×10^-3^, *η* ^2^: 0.41, 0.13, 0.13); and (ii) the main effect of age was significant for rotation amplitude, only (*F*=4.34, *p*=0.02, *η* ^2^ = 0.04), but there was no significant main effect of age for total rotation time and symmetry (*F*: 0.80, 1.89; *p*’*s*>0.05; *η* ^2^: 0.02, 0.04). In addition, there was no significant interaction between population-type and age for all the three metrics (*F*: 0.96, 0.66, 2.23; *p*’*s*>0.05; *η* ^2^: 0.02, 0.01, 0.04).

We carried out *post hoc* tests and found that: (i) Children with ASD had shorter total rotation time than TD children (*t*=-8.42, *p*<1×10^-3^, Cohen’s *d*=-1.63), as shown in **Figure 4A**; (ii) Children with ASD had weaker symmetry than TD children (*t*=-3.84, *p*<1×10^-3^, Cohen’s *d*=-0.74), as shown in **Figure 4B**; (iii) Children with ASD had lower rotation amplitude than TD children (*t*=-3.94, *p*<1×10^-3^, Cohen’s *d*=-0.76), as shown in **Figure 4C**; and (iv) ASD children aged 3.5-4.5 years had lower rotation amplitude than those aged 5.5-6.5 years (*t*=-2.91, *p*=0.01, Cohen’s *d*=-0.69, adjusted), but there was no significant difference of rotation amplitude in other pairwise comparisons, as shown in **Figure 4D**.

### 3.3 Classification

We constructed five classifiers using KNN, LDA, NB, SVM, and RF, and compared their performances using the ROC analysis. **Table 2** summarized our results, and showed that the NB classifier achieved the best performance with an accuracy of 88% and AUC value of 0.91. In addition, the performance ranking of the five classifiers may roughly be as follows: NB>SVM>RF>KNN>LDA.

**Table 2:** Quantitative evaluation results of prediction performance.

Classifier	Specificity	Sensitivity	Accuracy	AUC
KNN	0.90	0.80	85%	0.89
LDA	0.90	0.78	84%	0.90
NB	0.91	0.83	88%	0.91
SVM	0.89	0.81	85%	0.90
RF	0.85	0.84	85%	0.89

KNN, k-nearest neighbors; LDA, linear discriminant analysis; NB, naive Bayes; SVM, support vector machine; RF, random forest.

### 3.4 Correlation analysis

To examine the association between the three metrics and clinical (behavioral) measures using GDS-3, CARS, and PEP-3, we carried out a series of Spearman’s correlation tests for ASD participants. **Table 3** summarized our results and showed that: (1) Total rotation time positively correlated with PEP3-CVP, PEP3-EL, PEP3-RL, and PEP3-FM (*r*’s: 0.33~0.47, *p*’s<0.05); (2) Rotation amplitude positively correlated with PEP3-GM, Gesell-Ad-DQ, Gesell-GM-DQ, Gesell-FM-DQ, Gesell-La-DQ, and Gesell-PSB-DQ (*r*’s: 0.33~0.48, *p*’s<0.05); and (3) Symmetry negatively correlated with PEP3-CVP, PEP3-RL, PEP3-GM-DQ, Gesell-GM-DQ, and Gesell-FM-DQ (*r*’s: -0.35~-0.29, *p*’s<0.05); (4) PEP3-FM-DQ correlated with total rotation time (*r*=0.33, *p*<0.05); (5) PEP3-GM-DQ correlated with rotation amplitude and symmetry (*p*’s<0.05); (6) Gesell-FM-DQ correlated with rotation amplitude and symmetry (*p*’s<0.05); (7) Gesell-GM-DQ correlated with rotation amplitude and symmetry (*p*’s<0.05); (8) PEP3-VMI had no significant correlation with total rotation time, rotation amplitude, and symmetry (*p*’s>0.05); and (9) CARS score had no significant correlation with total rotation time, rotation amplitude, and symmetry (*p*’s>0.05).

**Table 3:** Spearman’s correlation analysis results.

Variables	Total rotation time	Rotation amplitude	Symmetry
PEP3-CVP	0.36*	0.18	-0.35*
PEP3-EL	0.47*	0.16	-0.30
PEP3-RL	0.39*	0.13	-0.36*
PEP3-FM	0.33*	0.23	-0.19
PEP3-GM	0.28	0.33*	-0.31*
PEP3-VMI	0.16	0.23	-0.18
Gesell-Ad-DQ	0.17	0.45**	-0.28
Gesell-GM-DQ	0.26	0.40**	-0.33*
Gesell-FM-DQ	0.17	0.41**	-0.29*
Gesell-La-DQ	0.28	0.48**	-0.20
Gesell-PSB-DQ	0.10	0.41**	-0.20
CARS score	0.12	-0.12	0.09

*: p<0.05; **: p<0.01; PEP3, Psychoeducational Profile-3; CARS, Childhood Autism Rating Scale; CVP, Cognitive Verbal/Preverbal; EL, Expressive Language; RL, Receptive Language; FM, Fine Motor; GM, Gross Motor; VMI, Visual Motor Imitation; DQ, developmental quotient; Ad, Adaptability; La, Language; PSB, personal-social behavior.

## 4 Discussion

Although children with ASD usually experience difficulties in meaningless GMI, few test paradigms have been devised. This study proposed a WRI task and established a WRI ability evaluation system using low-cost wearable inertial sensors. By this framework, three metrics (i.e., total rotation time, rotation amplitude, and symmetry) were extracted during the WRI task, which could be applied to reveal imitation impairments in children with ASD. In particular, our findings showed that children with ASD may exhibit decreased WRI abilities (evaluated by the three metrics). Furthermore, those decreases have been successfully applied to build ML-based classifiers in the current study. As far as we know, this is the first time to report such the concept and results, which illustrates a novel strategy for screening and diagnosis of ASD.

There are two distinctive kinds of GMI regarding the representational content of the observed actions (13). The first is the imitation of meaningless actions, for which an individual bypasses long-term memory and instead transforms visuospatial characteristics directly into motor representations (16, 43). This kind of imitation may activate brain regions belonging to the dorsal stream (16, 44). The second is the imitation of meaningful actions, for which an individual has a long-term memory template and thus can recognize the meaning or goal underlying the actions (43). This kind of imitation involves indirect semantic processing, which may activate brain areas belonging to the ventral stream (44). It is evident that WRI is essentially a meaningless GMI, does not rely on long-term memory, and may activate brain regions belonging to the dorsal stream (16, 44). Therefore, as verified in this study, WRI may be adopted as a potential assisted tool for ASD diagnosis.

Our findings (see **Figure 4**) showed that compared to TD children, those with ASD exhibited shorter total rotation time, lower rotation amplitude, and weaker rotation symmetry. Those decreases might be partially interpreted by the fact that children with ASD exhibit functional impairments in motor memory (45), motor planning (46), motor coordination (7, 47–50), and visual attention in imitation (16). In particular, a recent study showed that visual attention to movement area in children with ASD was positively related to imitation performance in meaningless gestures (16). This implies that children with ASD experience difficulties in directing and sustaining their visual attention to the movement area to perform the meaningless movements accurately (16). Our results also support this notion. Furthermore, visual attention in imitation and meaningless GMI might share the neural circuits associated with the dorsal stream (16, 44). Such the hypothesis might bring some new insights into the understanding of the meaningless GMI in individuals with ASD.

The Spearman’s correlation analysis results (see **Table 3**) revealed the association between WRI abilities and developmental levels (evaluated by GDS-3 and PEP-3 scales). In particular, the reliability and feasibility of our technique might be supported by the fact that two metrics (i.e., rotation amplitude and symmetry) had high correlations with the gross motor abilities (measured by the subdomain of GDS-3 and PEP-3 scales) and fine motor abilities (measured by the subdomain of GDS-3 scale). Language development has long been associated with motor development, particularly manual gesture (e.g., 51, 52). Our findings supported this notion, and found that (i) there is a significant correlation between total rotation time and expressive and receptive language skills (evaluated by the subdomain of PEP-3) and; and (ii) there is a significant correlation between symmetry and receptive language skills (evaluated by the subdomain of PEP-3). However, this study also found that the three metrics had no significant correlation with the visual-motor imitation (measured by the subdomain of PEP-3). This might be interpreted that our WRI task is fairly simple and cannot cover all visual-motor imitation skills that should be acquired for children with ASD. In addition, this study also showed that there is no significant correlation between the three metrics and the symptom severity (measured by the CARS score). This implies that these metrics cannot be applied to predict the symptom severity of ASD. Thus, it will deserve to suggest more complex GMI tasks or/and extract more powerful motor metrics for the prediction of ASD symptom severity.

Since children with ASD may usually exhibit impaired motor functioning, many researchers attempted to establish ML-based classifiers utilizing various kinematic features for screening and even diagnosis of ASD (4, 53–60). For instance, Zhao et al. (58) constructed five ML prediction models, in which kinematic features were extracted from a hand movement task. They showed that a maximum classification accuracy of 88.37% was reached with the k-nearest neighbor (58). Similarly, Zhao et al. (60) extracted kinematic features from head movement, and achieved a maximum classification accuracy of 92.11%. Remarkably, kinematic features from other motor tasks (e.g., head movement, touch-sensitive tablet interaction, reach-to-drop task, and imitation of hand movements) have also been investigated in the identifying of ASD (53, 54, 56). Some studies also aimed to reveal the stereotyped behaviors of ASD by kinematic features (54, 61). Our study suggested using the WRI task and extracting the three metrics, and verified that: (i) individuals with ASD exhibited WRI impairments; and (ii) classifier with the naive Bayes algorithm achieved a maximal classification accuracy of 88% and a maximal AUC value of 0.91. Remarkably, our method can be completed within two minutes, and may be more convenient and faster than existing techniques.

Strengths of the current study include: (i) the usage of a rapid rotation motion evaluation framework using low-cost wearable inertial sensors, (ii) the suggestion of three metrics correlated with developmental levels (evaluated by GDS-3 and PEP-3 scales), and (iii) the construction of a classifier with a classification accuracy of 88% and an AUC value of 0.91. However, there are also some limitations. First, in order to control the influence of sex, we exclusively invited boys to participate in our study. Even though some studies showed that sex composition did not significantly predict effect size (see 3 for a review), it is still necessary to test whether the inclusion of girls in the sample would have an impact on the current study’s findings. Second, we only invited children with IQ>70 to attend the current study because ASD children with IQ<70 may be difficult to imitate other’s motor behaviors, though our WRI task is very simple. It deserves to recruit the participants with IQ<70 and test the influence of IQ on the results. Third, we excluded TD participants with extremely low IQ levels only based on teachers’ reports, rather than conducting formal IQ tests on them. This limitation makes us unable to analyze whether IQ levels may have an impact on our findings. Fourth, participants from preschoolers were chosen in this study. This selection of participants prevents us from knowing whether our findings can also be applied to younger children with ASD. Therefore, it would be significant to extend the current technique to infants or toddlers (before 24 months) for early screening of ASD. Fifth and finally, we used the Gesell, PEP-3, and CARS scales to measure developmental abilities, and ASD symptoms, respectively. In future studies, it will be recommended to use ADOS-2 (62) or ADI-R (63) for describing the core symptoms of ASD, and use the Griffiths (64) or Mullen Scales of Early Learning (MSEL) (65) for evaluating developmental abilities.

## 5 Conclusion

We established a WRI ability evaluation system with low-cost wearable inertial sensors. Our findings showed that children with ASD may exhibit decreased WRI abilities. Those decreases have been successfully applied to construct classifiers using various ML algorithms. In particular, the classifier with the naive Bayes algorithm achieved a maximal classification accuracy of 88% and a maximal AUC value of 0.91. The high correlation of our method with gross motor and fine motor abilities (evaluated by the subdomains of GDS-3 and PEP-3) was observed. In addition, we also found that the suggested WRI task cannot cover all meaningless GMI domains and cannot predict the ASD symptom severity (measured by the CARS score). Given the strengths of the current study in terms of the low-cost and rapid measurement system, high consistency with clinical measures, and high classification accuracy, our WRI evaluation framework using low-cost wearable inertial sensors appears to hold promise as a rapid ASD screening approach from the data that currently exist.

## Data availability statement

The original contributions presented in the study are included in the article/supplementary material. Further inquiries can be directed to the corresponding author.

## Ethics statement

The studies involving humans were approved by The Ethics Committee of the Sanmenxia Center Hospital. The studies were conducted in accordance with the local legislation and institutional requirements. Written informed consent for participation in this study was provided by the participants’ legal guardians/next of kin. Written informed consent was obtained from the individual(s) for the publication of any potentially identifiable images or data included in this article.

## Author contributions

FL: Software, Writing – review & editing, Writing – original draft, Visualization, Validation, Resources, Methodology, Investigation, Formal analysis, Data curation. KQ: Software, Writing – review & editing, Writing – original draft, Visualization, Validation, Resources, Methodology, Investigation, Formal analysis, Data curation. HW: Software, Writing – review & editing, Writing – original draft, Visualization, Validation, Resources, Methodology, Investigation, Formal analysis, Data curation. YD: Writing – review & editing, Writing – original draft, Visualization, Validation, Resources, Methodology, Investigation, Formal analysis, Data curation, Conceptualization. DY: Supervision, Project administration, Funding acquisition, Writing – review & editing, Writing – original draft, Visualization, Validation, Resources, Methodology, Investigation, Formal analysis, Data curation, Conceptualization.
